# Teachers' motivational prosody: A pre‐registered experimental test of children's reactions to tone of voice used by teachers

**DOI:** 10.1111/bjep.12567

**Published:** 2022-12-04

**Authors:** Silke Paulmann, Netta Weinstein

**Affiliations:** ^1^ Department of Psychology, Centre for Brain Science University of Essex Colchester UK; ^2^ School of Psychology and Clinical Language Sciences University of Reading Reading UK

**Keywords:** education, motivation, prosody, self‐determination theory, teachers

## Abstract

**Background:**

Teachers' behaviours drive motivational climates that shape children's engagement and well‐being in the classroom, but few studies examine how specific teachers' behaviours such as wording, body language, or voice contribute to these outcomes in isolation of one another.

**Aims:**

This pre‐registered experiment sought to examine the often‐forgotten role that teachers' tone of voice plays in children's education. Informed by the theoretical framework of self‐determination theory (SDT; Ryan & Deci, *Self‐determination theory: Basic psychological needs in motivation*, *development*, *and wellness*, 2017), conditions manipulated controlling (pressuring, demanding), autonomy‐supportive (inviting of choice), or motivationally neutral, tones of voice to explore their effects on children's self‐reported psychological needs satisfaction, well‐being, intention to self‐disclose to and intention to cooperate with their teacher.

**Sample and Method:**

Children aged 10–16 years (*n* = 250) heard pre‐recorded teachers' voices holding sentence content and speakers constant across conditions, but varying tones of voice.

**Results:**

We hypothesized a‐priori and found that when children heard controlling sounding voices, they anticipated lower basic psychological need satisfaction, well‐being, and intention to disclose to teachers, as compared to neutral‐sounding voices. We also anticipated beneficial effects for autonomy‐supportive versus neutral voices, but pre‐registered analyses did not support these expectations. Intention to cooperate with teachers did not differ across conditions. Supporting relational motivation theory (RMT; Deci & Ryan, *Human Motivation and Interpersonal Relationships*, 2014), exploratory analyses showed that hearing autonomy‐supportive sounding voices increased autonomy and relatedness need satisfactions (but not competence need satisfaction), and through doing so indirectly related to beneficial outcomes (well‐being, intention to cooperate and self‐disclose).

**Conclusion:**

In summary, tones of voice seem to play an important role in shaping teachers' impact on their students.

## BACKGROUND

One of the most important tools in a teacher's toolkit is their voice: they use it for teaching or managing behaviour. Effectively using voice is crucial for pupils' educational and well‐being outcomes at school, and influences their information processing and comprehension (Rogerson & Dodd, 2005). For example, teachers who do not vary their tone struggle to keep pupils' attention (Schmidt et al., 1998), and teachers strategically use wider pitch variations to convey incorrect answers and help guide students to a correct one (Sikveland et al., 2021). However, we know little about the effects of inviting or demanding voices often heard in the classrooms. This is surprising given that it has been argued that teachers speaking in a warm “mellow” voice are regarded as more trustworthy while teachers speaking in a harsh sounding voice may be perceived as threatening (Martin & Darnley, 2017).

The current experiment set out to explore the role of tone of voice in teachers' communications through the motivational lens of self‐determination theory (SDT; Ryan & Deci, 2017), focusing on motivational sounding voices that deliver key feedback through autonomy‐supportive (supportive of children's voice and personal volition) or controlling (demanding or pressuring) tones. These qualities of teachers' communication are important to explore because teachers spend substantial time motivating students' behaviour, and the motivational quality as autonomy‐supportive or controlling they convey has implications for students' experiences and learning in the classroom (Niemiec & Ryan, 2009). To illustrate, a teacher who wishes his students to prepare for the next week's exam can warmly and gently (but still firmly) convey the importance of the preparations ahead, or otherwise express through a harsh or strict tone that students better be prepared. Autonomy‐supportive climates energize to action through a warm and collaborative climate where students are given choices about how to engage information, and where teachers make clear the usefulness of doing so. They are contrasted with controlling climates, in which teachers attempt to drive action through pressuring, judging performance and cooperation, and providing positive regard conditional on action (Black & Deci, 2000). Although the vast majority of this literature focuses on words and actions that create motivational climates, the example above illustrates how both words *and* tone may impact on students. Further, early work outside the education domain has also shown that these voice patterns can affect adolescents' behaviour (e.g., pressuring tones create rebellion; Weinstein et al., 2020), lower well‐being, and connectedness (Weinstein et al., 2018). Combined, these findings suggest that (in)adequate voice use can have profound effects. Interestingly, while the Teaching Agency (2012) has previously advised that “trainees should be able to vary the tone and volume of their voice to teach effectively and manage behaviour,” a systematic literature on the effects of voice in the classroom is still missing. Using the search engine Web of Science in the summer/autumn of 2022 (last search October 2022), we searched for relevant articles using the following keyword combinations “prosody and teaching” (257 entries); “prosody and education” 1(3 entries); “voice used in teaching” (833 entries). Title and abstract information were reviewed for all entries. While there is a sizeable literature exploring how prosody or voice can be used to teach oral skills, we only identified a handful of manuscripts addressing how voice can be used to effectively direct the classroom. The Web of Science search was supplemented with similar searches using PubMed and Google Scholar and we also explored potentially relevant teacher resources (e.g., The chartered College of Teaching; bera.ac.uk). All relevant literature identified in our search is cited in the current manuscript.

### Motivation in the classroom

The research builds on self‐determination theory (SDT; Ryan & Deci, 2017), a macro‐theory of human motivation that argues that motivation can be autonomous‐driven by personal valuing or interest, that is, from within the actor's self. Alternatively, motivation can be controlled‐driven by external demands, pressures, or consequences, that is, external to the self. In previous research with children and adolescents, controlling versus autonomy‐supportive social contexts have been shown to undermine well‐being in and out of the classroom (Niemiec & Ryan, 2009; Núñez & León, 2015). Furthermore, when exposed to controlling climates, youngsters and adolescents, in particular, have been shown to rebel rather than cooperate with adults (Van Petegem et al., 2015; Vansteenkiste et al., 2014), an intriguing outcome because it fundamentally undermines the aims of motivators, namely, to increase the desired behaviour. Finally, children and adolescents are less likely to intend to disclose personal experiences to controlling versus autonomy‐supportive parents (Tokić & Pećnik, 2011; Weinstein et al., 2021). Such lack of disclosure fundamentally undermines the ability of teachers and other responsible adults (i.e., caregivers) to care for and protect youngsters, and it leaves youngsters alienated and vulnerable (Boulton et al., 2013; Roth et al., 2009).

Despite the extensive body of work, this existing literature has three major limitations: First, existing work examining cooperation and self‐disclosure is largely, though not exclusively, in the context of parenting rather than education, and little is known about whether it would extend to teachers' influences on their students. Second, this research examines controlling and autonomy‐supportive contexts broadly, in terms of a myriad of behaviours and experiences that drive a global subjective perception by the youngster. Yet, motivational communications are also driven by specific factors within the interaction between teachers and students (e.g., body language, words, tone of voice), and these factors are largely ignored as drivers in their own right; thus, little is known about the role that tone of voice, specifically, plays. Finally, most research directly contrasts autonomy‐supportive to controlling motivational climates (Gerson et al., 2019; Weinstein et al., 2020), and few experimental studies examine both in relation to a neutral comparison (Paulmann et al., 2019; Weinstein et al., 2018) to determine which motivational climate drives beneficial or detrimental effects.

### Motivational prosody

Social environments can support autonomy or increase controlled motivation through the ways that teachers verbally communicate to their students (Reeve, 2011, 2012), but little work has been done isolating the role that tone of voice plays in this. Indeed, while teacher training has acknowledged the importance of voice use in the classroom, the recommendations put forward (e.g., an unforced voice creates warmth and is inviting the class to participate; a controlling voice lacks empathy; c.f. Martin & Darnley, 2017) are often based on anecdotal evidence rather than systematic research which we aim to provide here. Indirect evidence that teachers' voice matters come from previous work with adult participants that has shown that different voice patterns are used when creating particular motivational climates. For instance, when communicating autonomy‐support, speakers tend to use a quieter voice, slower speech rate and less voice energy (making the voice sound “soft”) compared to when they are expressing controlling vocalizations, which are characterized by an increase in voice energy used (making the voice sound “harsher”; Weinstein et al., 2018). In addition to acoustic differences, the two types of motivational voices have also been shown to recruit different neural networks in listeners (Paulmann et al., 2019). Specifically, past research that directly compared autonomy‐supportive and controlling prosody reported a differentiation between the two motivational intentions in event‐related brain potentials (P200, late potential) that were also differently distributed. Finally, the two types of motivational voices have also been shown to affect social ties (Weinstein et al., 2018).

In laboratory studies with adult participants, listening to controlling voices increased anticipated defiance to requests and decreased intended cooperation, whereas the opposite pattern was observed for autonomy‐supportive sounding voices (Weinstein et al., 2019). The only work sampling adolescents of which we are aware found that hearing parents' controlling motivational voices undermines youngsters' well‐being and reduces their intention to cooperate with their parents (Weinstein et al., 2020). Similarly, in an early study by Deci et al. (1993), 6‐ to 7‐year‐old children's intrinsic motivation was negatively correlated with controlling vocalizations (though note that controlling intentions were communicated through both sentence content and voice cues used, thus making it difficult to judge the individual contributions of each channel to the outcome). Taken together, these data suggest that motivational voice usage can impact youngster's well‐being and behaviour.

In a teaching context, findings summarized above should be of particular interest given a different line of research that explored the effects of voice use on information retention. This limited research suggests that children will not learn as effectively if spoken to in a voice that is considered inappropriate by them (e.g., negative tone used when delivering instructions) or that sounds unpleasant and is unpreferred (Gampel & Ferreira, 2017; Martin & Darnley, 2017; Schmidt et al., 1998). Furthermore, teaching with impaired voices (e.g., hoarse, weak, strained) reduces how well children process information (Rogerson & Dodd, 2005). Voice impairment related to pitch, volume, tone, and voice quality has been linked to continuous straining of voices, such as when teachers try to maintain discipline in a noisy classroom by shouting over the noise level (c.f. Rantala et al. (2015) for evidence that teachers speak habitually louder when they are teaching in noisy environments). In short, there is accumulating evidence that more research on teachers' voice usage and its effects on students is well‐warranted. The current investigation aims to help close this gap in the education literature.

#### Motivational prosody and psychological need satisfaction

A proximal reason for motivational prosody effects on child outcomes is that such tones of voice can influence children's psychological need satisfaction. SDT argues three basic psychological needs underlie well‐being and drive positive learning and interpersonal behaviours, including in the classroom. First, autonomy satisfaction is the experience that one is choiceful, volitional, and able to have a voice. Second, relatedness is the feeling that one is close and connected to others, including to teachers. Finally, competence is the feeling that one can achieve desired goals and engage in tasks effectively (Reeve, 2012; Ryan & Deci, 2016; Wang et al., 2019). Previous research in educational contexts shows that controlling versus autonomy‐supportive teaching styles thwart the three basic psychological needs. Through this, these teaching styles undermine students' well‐being, engagement, and positive relationships with teachers (Hein et al., 2015; Liu et al., 2017; Van den Berghe et al., 2013). As such, psychological need satisfaction has been modelled as a proximal outcome of motivational climates, which bears on downstream intrapersonal and interpersonal consequences. Similarly, Weinstein et al. (2020) identified that basic psychological need satisfaction mediated the effects of motivational prosody on well‐being outcomes of adolescents after hearing parents' voices.

### Present experiment and rationale

We designed our pre‐registered analytic plans to test three downstream outcomes of motivational tones of voice: well‐being, self‐disclosure intention, and cooperation. These outcomes were selected because they have been the focus of previous investigation of adolescent‐adult motivational communications. We operationalized well‐being in line with previous research showing that supportive motivational communications are particularly important for this construct when measured in terms of positive emotions and greater self‐esteem, as well as the absence of negative emotions (Weinstein et al., 2018), including in adolescent samples (Weinstein et al., 2019). We also focused on self‐disclosure intention because previous research has shown supportive adolescent‐adult communications are important for the felt security within relationship that promote the willingness to further disclose events (Weinstein et al., 2021), which allow teachers to protect kids from experiences such as bullying (Macaulay et al., 2018) and self‐harm (Freedenthal & Breslin, 2010). Moreover, we sought to test a motivationally‐specific outcome, and selected cooperation as a proximal indicator that reflects the extent that motivating requests made by teachers would be likely to elicit their desired actions (Hsu et al., 2021; Koh & Frick, 2010).

Finally, as this is the first study to systematically explore the effects of motivational prosody on children in a school setting, we wanted to assess the influence across different adolescent ages to infer generalizability of effects. We thus followed previous research (Nitsch et al., 2015; Richaud et al., 2013; Steinberg & Silverberg, 1986) who also sampled a wide age range of adolescents from early years (aged 10) to late adolescents (16‐year‐olds). Indeed, past work has highlighted that this is a time when children balance growing independence with dependence on adults (Steinberg & Silverberg, 1986).

### Hypotheses

Pre‐registered hypotheses (https://osf.io/j8ywq/?view_only=91f63e464b0a4134b9d9e7898c75194b) concerned the main and mediating effects of the motivational prosody manipulation. Hypothesis 1–4 anticipated that condition would predict each of four tested outcomes. Specifically, we expected that conditions would predict:

Hypothesis (1) Psychological need satisfaction (autonomy, relatedness, competence).

Hypothesis (2) Well‐being (positive affect, negative affect (r), self‐esteem).

Hypothesis (3) Self‐disclosure intention.

Hypothesis (4) Intention to cooperate.

Following this, we anticipated that the autonomy‐supportive condition would predict greater scores than the neutral condition, and the controlling condition would predict lower scores than the neutral condition.

Hypothesis 5 further anticipated that psychological need satisfaction would mediate the effect of condition on downstream outcomes (well‐being, future self‐disclosure, and intention to cooperate).

#### Transparency and openness

We report how we determined our sample size, all data exclusions, all manipulations, and all measures in the study, and we follow JARS (Kazak, 2018). All data, analysis code, and research materials will be made available on our OSF link upon publication. Data were analysed using SPSS version 27.

## METHOD

### Ethics

The study has received ethical approval through the Ethics Subcommittee 3 of the University of Essex (ETH2021‐1238).

### Participants

Two‐hundred fifty children based in the UK aged 10–16 years participated in an online study. They were recruited in collaboration with Childwise, a panel company working with schools and communities. The planned sample size of *n* = 250 was set to achieve 95% power to detect a medium *f* coefficient = .25 for the omnibus effect of three conditions, at *α* = .05. The sample was composed of 116 (46.4%) boys and 133 (53.2%) girls; 1 child reported another gender. Participant ages ranged from 10–16 years with *M* = 12.18 *SD* = 1.89 (10.8%–18.8% of the sample fell into each year of age).

### Stimuli development and validation

Auditory samples were pre‐recorded by teachers and validated with independent samples, through a process described in detail in Appendix S1 (OSF).

#### Surveys

##### Perceived autonomy‐supportive and controlling climate

The manipulation check was measured using two items adapted from Weinstein et al. (2021) who used them in a study of perceived support with mid‐ and older adolescents. Participants were asked: “If my teachers asked me to complete a school activity using this tone of voice I would… feel that my teacher shows interest in me and is willing to listen” (perceived autonomous climate), and “feel that my teacher is pressuring or forcing me to behave a certain way” (perceived controlling climate). The two items correlated *r* = −.22, *p* = .001. Items were paired with a scale from “a great deal” (coded 5) to “not at all” (coded 1).

##### Psychological need satisfaction

The basic psychological need satisfaction scale (La Guardia et al., 2000) is often used in close relationships and has been used as a predictor of positive and negative affect in emerging adults, showing internal reliabilities of *α* = .60–.90, and responding in theory‐consistent ways to autonomy‐supportive climates (Şimşek & Demir, 2013; Weinstein et al., 2021). It was adapted to the teaching domain in this age range. Participants responded to the stem “Imagine spending the school day with a teacher who spoke like this. How much would you feel that you…”. Items for autonomy need were as follows: “are free to be you,” “can share your opinion,” or “feel pressured and pushed around” (*α* = .61). Items for relatedness were “are cared about,” “feel close to your teacher,” “feel distant from your teacher” (*α* = .66). Items for competence were “are good at what you do” and “feel that you can do things well” (*α* = .88). A deviation from pre‐registration, a third item: “are not good at activities,” was removed because it brought *α* to .44 (with corrected item‐total correlation = −.08).^1^ Items were paired with a scale from “a great deal” (coded 5) to “not at all” (coded 1). An exploratory factor analysis showed that three subscales loaded on one principal component together, which accounted for 86.2% of the variance; higher‐order reliability was *α* = .92 for the need satisfaction composite.

##### Well‐being

A well‐being composite was composed of positive and negative affect (reversed) from the positive and negative affect schedule (PANAS; Watson et al., 1988). Participants responded to the stem: “how much would a teacher who spoke like the examples make you feel…” “happy,” “pleased,” “interested” (6 items, *α* = .97), and negative affect: “afraid,” “miserable,” “ashamed” (6 items, *α* = .94). In addition, participants reported on their self‐esteem with a validated face‐valid single‐item measure used with children “I would have high self‐esteem,” which has shown highly similar predictive patterns with the more widely used Rosenberg Self‐Esteem inventory (Robins et al., 2001). Higher‐order reliability was high, and the three subscales were combined as was planned prior to the study. Items were paired with a 7‐point scale from “extremely” (coded 7) to “not at all” (coded 1; *α* = .66). An exploratory factor analysis showed that three subscales loaded on one principal component together, which accounted for 61.8% of the variance; higher‐order reliability was *α* = .66 for the full composite. This composite was consistent with previous research that has combined these indicators for a well‐being measure in emerging adults aged 17–21 in the context of cyberbullying (Waisglass, 2017), as a result of parental listening (Weinstein et al., 2021), and in relation to daily experiences following social media use (Mitev et al., 2021).

##### Self‐disclosure intention

Self‐disclosure intention was adapted from Miller et al. (1983). Participants responded to the stem: “Imagine spending the school day with a teacher who spoke like this. How likely is it that you would tell your teacher…” with five items that measured their intention to disclose both positive and negative experiences: “if you were bullied,” “what your interests are,” “things you have done which you are proud of,” “an embarrassing event,” “something that worries you.” A similar measure was used previously to measure intention to disclose to parents after a vignette paradigm that manipulated listening in an adult sample (Weinstein et al., 2021). Items were paired with a scale from “extremely likely” (coded 5) to “extremely unlikely” (coded 1; *α* = .92).

##### Cooperation intention

Cooperation intention was constructed from four items that tapped at different facets of the construct, taken from Weinstein et al. (2020): “be inclined to do the exact opposite of what my teacher wanted me to” (r), “commit myself to what my teacher asked and cooperate,” “put a lot of effort into what my teacher asked,” and “listen carefully.” Items were paired with a scale from “a great deal” (coded 5) to “not at all” (coded 1; *α* = .63).

##### Design and procedure

After consenting online, participants reported their age and gender, and received information they would engage in a task to listen to a number of phrases. Specifically, they were told “Next, you will be asked to listen to recordings of teachers saying different things. These teachers are saying things you may hear in school. When you listen, imagine that the teachers are talking to you personally. Try to focus on how they are talking to you – think about how you feel or what you would think. To help you imagine the situations better, we will play the same sentences several times. Please make sure your volume is turned up.” Participants were fully randomly assigned to receive one of three between‐subject conditions: controlling sounding voice, neutral‐sounding voice, and autonomy‐supportive sounding voice. All listened to a clip, varying by condition assignment but lasting 80 seconds, and confirmed they heard it (this was further checked by looking at timings between when the audio started to play and participants confirming they heard the sound files). They were then asked to report on their reactions and expectations based on the clip they heard, with the initial prompt “The next few pages will ask you to imagine a teacher who sounds like the ones you just heard.” These instructions have been used in similar experimental paradigms (e.g., using vignettes, or proxy stimuli) to enhance the depth of experience and specificity of reactions to the manipulation (Weinstein & Przybylski, 2019). Surveys were presented in a fully randomized order in case of survey carry‐over or reduced attention throughout the remainder of the study.

##### Pre‐registered plan

Prior to conducting the study, we pre‐registered our hypotheses, design, and analytic plan on OSF (Weinstein & Paulmann, 2021). We anticipated we would exclude those who fall outside our anticipated age ranges, who do not pass the audio screener, or who cannot hear the stimuli. A notable deviation from our pre‐registered plan is that following advice from Childwise, we also excluded participants who completed the study – including both parent and child consent procedures, stimuli, and surveys in fewer than 3.5 min, that is in 50% of the time (7 min) that we and Childwise had anticipated the study should take. This time period would have meant they had a maximum of one minute to complete all our surveys. 27 participants were excluded for this reason and replaced by Childwise for a total of *n* = 250 as planned. All other analytic activities followed the pre‐registered plan, unless described in the text below.

## RESULTS

### Analytic approach

We planned to use a between‐subjects multiple analysis of variance (MANOVA) to test for significant condition effects across all outcomes tested. The MANOVA's omnibus effect allowed us to check that condition impacted outcomes sufficiently robustly across the multiple tests conducted. As a first step, we checked and observed dependent variables met assumptions of normality and found that none of the dependent variables tested showed skewness or kurtosis characteristics that violated normality assumptions (>1.0; Hair et al., 2022). Further, sample sizes were independent and equal across conditions to protect against heterogeneity of variance (Parra‐Frutos, 2013).

### Pre‐registered main effects

We pre‐registered that we would test main effects hypotheses using between‐subjects analysis of variance (ANOVA), with conditions defined as a predictor. We followed this plan through a MANOVA, which also allowed us to test support for an omnibus effect of condition across all the outcome variables tested, and minimize the likelihood of spurious effects for a small number of outcomes. Results of MANOVA showed an overall effect of condition across all outcomes, *F*(12, 484) = 4.40, *p* < .001, *d* = .08.

Examining outcomes separately, main effects of condition were present predicting psychological need satisfaction, well‐being, and self‐disclosure intention. These results are summarized in Table 1. Those assigned to the Controlling voice condition reported lower need satisfaction, *t*(166) = −2.67, *p* < .001, *d* = *−*.41, lower well‐being, *t*(166) = −3.92, *p* < .001, *d* = *−*.61,^2^ and lower self‐disclosure intention, *t*(166) = −2.71, *p* < .001, *d* = *−*.42; controlling tones undermined their psychological needs, increased their negative relative to positive emotions, lowered their self‐esteem, and dissuaded youngsters from seeking to share their triumphs and problems with that teacher in the future.

**TABLE 1:** Main effects and post‐hoc comparisons contrasting autonomy‐supportive, controlling, and neutral conditions on all study outcomes

	*F* omnibus	Aut *M* (*SE*)	Neut *M* (*SE*)	Cont *M* (*SE*)	Controlling vs. neutral			Autonomy‐supportive vs. neutral			Autonomy‐supportive vs. controlling		
					*t*	*p*	*d*	*t*	*p*	*d*	*t*	*p*	*d*
Pre‐registered											Exploratory		
Perceived controlling climate	8.42	2.81 (.14)	3.04 (.14)	3.58 (.14)	2.81	.005	.44	−1.17	.242	−.19	3.99	<.001	.62
Perceived autonomous climate	2.22	3.01 (.15)	2.98 (.15)	2.62 (.15)	−1.73	.085	−.27	.17	.861	.02	−1.89	.058	−.29
Need satisfaction	10.51**	3.18 (.11)	2.89 (.11)	2.49 (.11)	−2.67	<.001	−.41	1.89	.059	.29	−3.90	<.001	−.61
Well‐being	13.83**	4.08 (.12)	3.89 (.12)	3.23 (.12)	−3.92	<.001	−.61	1.07	.286	.17	−4.99	<.001	−.78
Self‐disclosure intention	8.90**	3.22 (.12)	2.99 (.12)	2.54 (.12)	−2.71	<.001	−.42	1.43	.154	.22	−4.15	<.001	−.64
Cooperation intention	1.63	3.31 (.10)	3.43 (.10)	3.18 (.10)	−1.80	.072	−.28	.82	.410	.13	−.98	.329	−.15
Exploratory													
Autonomy	12.58**	3.21 (.11)	2.91 (.11)	2.46 (.11)	−2.95	<.001	−.46	2.02	.044	.31	−4.99	<.001	−.78
Relatedness	11.50**	3.12 (.11)	2.80 (.11)	2.39 (.11)	−2.73	.007	−.42	2.03	.042	.31	−4.78	<.001	−.74
Competence	5.03**	3.22 (.13)	2.97 (.13)	2.63 (.13)	−2.71	<.001	−.42	1.43	.154	.22	−3.16	.002	−.49

*Note*: The table lists statistical results for all pre‐registered outcomes. All pairwise comparisons were based on directional hypotheses. **p < .001.

Abbreviations: Aut, participants listened to autonomy‐supportive sounding voices; Cont, controlling sounding voices; Neut, neutral‐sounding voices.

Condition did not predict intention to cooperate, *F*(247) = 1.63, *p* = .197. Further, the autonomy‐supportive voice condition did not predict higher standing on any of the outcomes measured, *t*s < 1.90; *p*s > .154; *d*s < .30),^3^ though the test for the psychological needs variable showed a trend in that direction, *t*(165) = 1.89, *p* = .059, *d* = .22. In sum, pre‐registered main effects analyses partially supported Hypotheses 1–3 showing that motivational voice effects were driven by Controlling voices, but we did not find support for Hypothesis 4.

### Pre‐registered indirect effects

Informed by the pattern of pre‐registered main effects, PROCESS (Hayes, 2012) was used to test indirect effects for the Controlling versus Neutral voice condition predicting outcomes through psychological need satisfaction. These results are summarized in Table 2. Findings showed an indirect effect through psychological need satisfaction was present linking condition effects to well‐being, *b* = −.51, *SE* = .14, 95% CI [−.79, −.26], intention to self‐disclose, *b* = −.45, *SE* = .12, 95% CI [−.69, −.22], and intention to cooperate, *b* = −.29, *SE* = .08, 95% CI [−.45, −.14]. When accounting for these indirect effects, the previously significant direct effect of condition on intention to self‐disclose was no longer significant, *b* = −.11, *SE* = .10, 95% CI [−.30, .08], though the previously significant direct effect on well‐being remained significant, *b* = −.25, *SE* = .14, 95% CI [−.79, −.26]. In summary, results of indirect effects analyses showed that controlling voices undermined well‐being, inhibited future self‐disclosure, and dissuaded cooperation through its effects on basic psychological need satisfaction anticipated by students.

**TABLE 2:** Indirect effects of condition on each of the three study outcomes (well‐being, intention to disclose, and intention to cooperate) through basic psychological needs

Predictor	Mediator	Outcome	Indirect effect			
			*b*	*SE*	95% CI −	95% CI +
Pre‐registered						
Controlling vs. neutral	All needs	Well‐being	−.51	.14	−.79	−.26
Controlling vs. neutral	All needs	Intention to disclose	−.45	.12	−.69	−.22
Controlling vs. neutral	All needs	Intention to cooperate	−.29	.08	−.45	−.14
Exploratory						
Autonomy vs. neutral	Autonomy & relatedness	Well‐being	.50	.12	.26	.74
Autonomy vs. neutral	Autonomy & relatedness	Intention to disclose	.44	.11	.23	.66
Autonomy vs. neutral	Autonomy & relatedness	Intention to cooperate	.29	.07	.16	.44

*Note*: Predictor refers to the contrast code between each motivationally laden condition and the Neutral comparison condition. In the first contrast, the Controlling Prosody condition was coded 1 and the Neutral Prosody condition was coded 0. In a second contrast, the autonomy‐supportive Prosody condition was coded 1, and the Neutral Prosody condition was coded 0. All needs refer to all three basic psychological needs (autonomy, relatedness, and competence), defined as one composite following registered plans. Autonomy and relatedness were combined for one composite in exploratory analyses.

### Exploratory analyses

#### Which psychological needs drive effects?

Although we pre‐registered we would test psychological need satisfaction as a composite following previous conceptual and empirical practices to view the needs in sum (Ryan & Deci, 2000), autonomy‐supportive and controlling motivational prosody may influence the three psychological needs separately, and may particularly influence autonomy need satisfaction because it is directly intended to support this psychological need (Ryan et al., 2006), and relatedness, because it is a definitionally interpersonal process (Deci & Ryan, 2014). According to a more recent theory within SDT – Relationship Motivation Theory (RMT; Deci & Ryan, 2014) when autonomy is supported, relatedness is inevitably encouraged as well. As such, exploratory (unplanned) analyses considered the effect of condition on each of the psychological needs, separately. Results of a MANOVA analysis showed an overall omnibus effect of condition predicting greater autonomy need satisfaction, *F*(2, 247) = 12.58, *p* < .001, *d* = .09, relatedness need satisfaction, *F*(2, 247) = 11.50, *p* < .001, *d* = .09, and competence need satisfaction, *F*(2, 247) = 5.03, *p* = .007, *d* = .04.

Post‐hoc comparisons revealed that the Controlling versus Neutral voice contrast predicted the psychological need satisfaction for autonomy, *t*(166) = −2.95, *p* < .001, *d* = *−*.46 relatedness, *t*(166) = −2.73, *p* < .001, *d* = *−*.42, and competence, *t*(166) = −2.71, *p* < .001, *d* = *−*.42.

**FIGURE 1:**
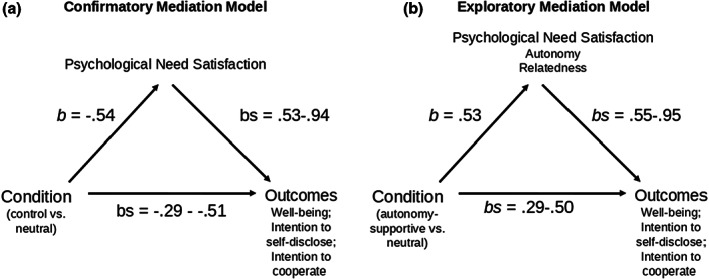
Confirmatory (a) and exploratory (b) mediation models that summarize indirect effects.

Moreover, examining the psychological needs separately, listening to autonomy‐supportive voices predicted greater autonomy, *t*(165) = 2.02, *p* = .044, *d* = .31, and relatedness, *t*(165) = 2.03, *p* = .042, *d* = .31, but did not increase competence, *t*(165) = 1.43, *p* = .152, *d* = .22 need satisfaction. Following on this divergence from the effects of the autonomy‐supportive condition when all three needs (including competence) were averaged, we conducted indirect effects in PROCESS wherein the autonomy‐supportive versus Neutral contrast predicted outcomes through only autonomy and relatedness need satisfactions. Findings showed an indirect effect through autonomy and relatedness need satisfaction was present predicting well‐being, *b* = .50, *SE* = .12, 95% CI [.26, .74], intention to self‐disclose, *b* = .44, *SE* = .11, 95% CI [.23, .66], and intention to cooperate, *b* = .29, *SE* = .07, 95% CI [.16, .44]. In sum, the autonomy‐supportive voice condition predicted greater anticipated outcomes for adolescents indirectly, through its beneficial effects on both autonomy and relatedness need satisfaction (Figure 1).

## DISCUSSION

This study set out to investigate how teachers' voice usage might affect students, an idea that has been discussed frequently amongst voice and teacher trainers, but which has not been explored systematically in the literature. The primary findings following a pre‐registered analytic plan showed that the use of controlling voices (vs. autonomy‐supportive voices) drove effects. When children heard teachers use controlling tones of voice that were pressuring or demanding, they expected to feel lower psychological need satisfaction, lower well‐being in terms of affect and self‐esteem, and less intention to disclose personal information to those teachers in the future. These findings are consistent with past electrophysiological work that shows that controlling voices, more so than autonomy‐supportive voices, command immediate attention when compared to neutral‐sounding voices (Paulmann et al., 2019; Zougkou et al., 2017). Here, any attentional preferences to demanding sounding voices observed in previous research were detrimental, not beneficial, to our participants. We found moderate effect sizes of controlling voices reducing children's anticipated well‐being, despite a relatively brief (80 s) exposure, highlighting how powerful voice cues can be in the classroom (in which teachers often speak for about 60% of the school day; Martin & Darnley, 2017). These results also suggest that previous findings linking the harmful effects of controlling teaching styles to students' well‐being (Grolnick & Ryan, 1987; Ryan & Deci, 2009) could in part have been influenced by the way that teachers utilized their voices.

Following the theoretical approach of SDT, our most immediate hypothesized outcome was satisfaction of the psychological needs for autonomy, feeling choiceful and volitional in one's actions and free to self‐express, relatedness, feeling close and connected to others, and competence, feeling effective in one's behaviour. We based our expectations at the outset of the study on an extensive SDT literature demonstrating that autonomy‐supportive social contexts enhance psychological need satisfaction, while controlling social contexts thwart these needs (reviewed in Ryan & Deci, 2017). Additionally, a small handful of previous experimental studies have shown that motivational prosody affects downstream outcomes (such as well‐being) through its immediate effects on psychological need satisfaction in adults (Weinstein et al., 2018) and children responding to parental voices (Weinstein et al., 2020). Interestingly, exploratory analyses looking at needs separately showed that controlling sounding voices thwarted all three basic psychological needs consistent with previous research (Weinstein et al., 2020), but voice signalling autonomy support only satisfied the autonomy and relatedness need satisfaction. This pattern of finding for autonomy‐supportive sounding voices is consistent with relational motivation theory (RMT; Deci & Ryan, 2014), which argues that autonomy and relatedness needs, in particular, underlie meaningful interpersonal connections. In this experiment, controlling sounding voices undermined this connection, and the sense that students would feel competent in the school environment, whereas autonomy‐supportive teacher voices enhanced the anticipated interpersonal connection with teachers.

Controlling voices also dissuaded children from intentions to share secrets with their teachers, including positive ones that involved their achievements and negative ones such as being bullied. Children's self‐disclosure is a fundamental tool used by caring adults to intervene with, support, and guide children (Boulton et al., 2013; Roth et al., 2009), yet up to 80% of students do not believe or are not sure whether their teachers are interested in taking action to intervene with prominent school experiences such as bullying (Rigby & Bagshaw, 2003), and children often fail to disclose personal but relevant experiences at school (Fisher & Larkin, 2008). These findings here suggest that controlling sounding teachers can further interfere with this process and dissuade important self‐disclosures that would ultimately benefit both teachers' ability to effectively care for students and the students themselves.

Unlike in previous research testing motivational voices expressed by parents (Weinstein et al., 2020), we found little evidence that tone of voice directly changed intention to cooperate. Discrepancy in these findings speaks to a distinct possibility that motivational voices have somewhat different impacts across the domains of children's lives. It may be that when in school, children are socialized by peers and context to respond to teachers, especially when they are using controlling sounding voices. Indeed, many teachers report using firm and harsh sounding voices when managing behaviour in classrooms (c.f. Martin & Darnley, 2017), suggesting that the use of these vocal patterns is not restricted to isolated events but may be quite common in schools. As such, while children may defy and refuse to cooperate with parents communicating with control (Weinstein et al., 2020), teachers may achieve some cooperation even when using controlling sounding voices. Importantly, while we did not find that this voice usage undermined teachers' efforts, it was also not instrumental to achieving them. Taking our findings in sum, controlling sounding voices, in comparison with neutral ones, failed to achieve what they set out to do – ensure the child's compliant behaviour – while also producing unintended costs in the form of undermined psychological needs, well‐being and children's willingness to self‐disclose.

Across our outcomes, pre‐registered models showed that psychological need satisfaction mediated downstream detrimental effects of hearing controlling tones, and beneficial effects of hearing autonomy‐supportive tones. These findings supplement previous research showing that perceptions of teachers' autonomy‐supportive versus controlling behaviours increase engagement (Cohen et al., 2020; Zhou et al., 2019), vitality and educational satisfaction (Alp et al., 2018) *because* they support basic psychological need satisfaction in the classroom. The mediational model further lends evidence to an SDT view that positive motivation and well‐being in the classroom are borne of supportive climates that satisfy the three basic psychological needs (Ryan & Deci, 2009, 2016).

### Limitations of the study

While current findings largely support effects of controlling voice use reported in other domains and with other age groups (Weinstein et al., 2018, 2020), this vignette‐paradigm study should be replicated in the classroom. For example, the effects of teachers' voice on students' well‐being and behaviour could be measured by recording listening, engagement, and cooperation during particular school activities when teachers are more likely to attempt to motivate behaviour, and therefore may naturally gravitate towards autonomy‐supportive or controlling voices. This may involve instances as children move from one activity to the next (lesson to lunch), or during discussions of homework assignments. Motivational styles have also been quantified through coding videotaped recordings of teachers' behaviours (De Meyer et al., 2014) or through direct observations of teachers (Cents‐Boonstra et al., 2021). Such methods would be extremely useful here, with raters focused on acoustic and subjective characteristics of tone of voice used by teachers to drive behaviour.

Second, children's outcomes were assessed with self‐reports, but studies could evaluate the effects of voice on students using (electro)‐physiological markers (Paulmann et al., 2019) to gather more objective measures on well‐being and implicit processing of voice cues. Moreover, measuring the influence of voice use on academic outcomes similar to what has been reported in the emotional literature, would be beneficial. For example, past research suggests that children remember academic content better if they are feeling happy versus neutral during the encoding stage (Erk et al., 2003). Building on the SDT literature, which has shown that autonomy‐supportive practices create better learning environments, prosody use alone may help to establish such environments and help children perform better. This might be of special relevance for children with special educational needs or those coming from socially disadvantaged backgrounds as evidence revealed that the latter group is particularly sensitive to the way teachers speak in the classroom (Kashinsky & Wiener, 1969). Finally, while the current evidence supports the idea that voice use of teachers can help create autonomy‐supportive climates, our focus on psychological needs and well‐being of students does not provide direct evidence that different use of voices can also increase academic performance. Although past research has highlighted that voice dysphonia can impact academic performance (Rogerson & Dodd, 2005), it has not yet been tested if instructing students in autonomy‐supportive voices leads to better educational outcomes. Future studies should test this hypothesis.

### Next steps: practical guidance

Teachers speak for long periods of the day, often in noisy classrooms with bad acoustic set‐ups. The profession is known to have high prevalence rates for voice disorders (Roy et al., 2004); perhaps not surprisingly, teachers with impaired voices tend to leave the profession, contributing to low retention rates in educators as teachers with voice disorders are reported to experience lower job satisfaction than those without voice problems (De Alvear et al., 2010). Voice disorders in teachers also have an effect on children's learning as listening to impaired voices caused by inadequate voice usage reduces how well students process information (Rogerson & Dodd, 2005). Finally, working with voice disorders can increase experienced stress in teachers as evidenced in a study in Spain which reports that teachers with voice disorders were more likely to “sleep uneasily,” feel “irritable” and “overloaded” compared to those without voice disorders (De Alvear et al., 2010). It is therefore crucial that we develop a systematic and holistic understanding of how and which instructor voices create effective and positive learning environments without putting strain on voices. Creating a resilient repertoire that will equip teachers to support both the general population and disadvantaged children, enhance learning environments, and cope under stress is important for teaching quality as much as for creating supportive learning environments. Yet, teacher training is not compulsory as part of teacher education in most countries, and even if offered the delivery is not evidence‐based. This research took a first step at addressing an understudied area in education: how tone of voice is used to relate to, and influence children. While future work will be needed to extend our understanding of these processes in real‐world educational settings, the current pre‐registered findings suggest detrimental effects of teachers' controlling tones of voice on students, and exploratory findings suggest there may be more subtle relational benefits when teachers use autonomy‐supportive tones. Practically speaking, teachers may wish to enhance their classroom communication by thinking about the way they modulate pitch, rhythm or loudness. For instance, teachers who are aware that vocal qualities such as breathiness or creakiness or speech that sounds particularly throaty can create the impression of sounding harsh can invest in vocal hygiene or voice training that will help them avoid displaying such characteristics. Instead, focusing on sounding warm and supportive will help teachers create learning environments that support students' psychological need satisfaction and well‐being.

## AUTHOR CONTRIBUTIONS

**Silke Paulmann:** Conceptualization; data curation; funding acquisition; investigation; methodology; project administration; resources; software; writing – original draft; writing – review and editing. **Netta Weinstein:** Conceptualization; data curation; formal analysis; investigation; methodology; project administration; resources; software; writing – original draft; writing – review and editing.

## CONFLICT OF INTEREST

The authors have no conflict of interest to declare.

## FUNDING INFORMATION

This work was funded by an ESRC Impact Acceleration Account Fund FNA‐16 (DG02404) awarded to Silke Paulmann.

### OPEN RESEARCH BADGES

This article has earned Open Data, Open Materials and Preregistered Research Design badges. Data, materials and the preregistered design and analysis plan are available at https://doi.org/10.17605/OSF.IO/J8YWQ.

## Supporting information


Appendix S1.

## Data Availability

All materials and data will be uploaded to OSF upon acceptance for publication.

## Footnotes

^1^
These scales were designed to assess complex interpersonal constructs efficiently (with relatively few items per facet of a construct) and we were therefore comfortable with the lower but acceptable reliability above .60 (Nunnally & Bernstein, 1994). However, there was no apparent correlation between the two positively framed competence items, and the negatively worded ones. It may be the phrasing was confusing to participants in this study, though previous research using these items with adolescents found acceptable internal reliability across the three items (Weinstein et al., 2021). Alternatively, competence and incompetence (i.e., positively and negatively worded items) may have a different relationship in educational contexts than the previous parenting ones where these items were assessed; for example, a student may feel both that they are ‘good at what they do at school’, but when prompted consider they do not do certain activities particularly well. Further research examining the relation between competence and incompetence in relation to global school functioning or specific school activities would help to clarify whether different or expanded measures of competence would be more appropriate in this setting.

^2^
Given that our well‐being indicators of positive affect, negative affect, and self‐esteem are often treated as separate indicators in diverse research areas, we conducted an exploratory MANOVA wherein condition predicted these three outcomes separately. Results showed an effect of the Controlling voice condition predicting less positive affect, *F*(1, 247) = 12.61, *p* < .001 (*M*_control_ = 3.63; *M*_neutral_ = 4.30); more negative affect, *F*(1, 247) = 9.78, *p* = .002 (*M*_control_ = 4.13; *M*_neutral_ = 3.23); and less self‐esteem, *F*(1, 247) = 4.32, *p* = .039 (*M*_control_ = 2.44; *M*_neutral_ = 2.87).

^3^
Exploratory analyses similarly did not show an effect of the Autonomy‐supportive voice condition predicting positive affect, negative affect, or self‐esteem when defined separately, *F*s(1, 247) = 2.92, *p* = .089.
