# Supplementary material for: Teachers' motivational prosody: A pre‐registered experimental test of children's reactions to tone of voice used by teachers
Source: Br J Educ Psychol. 2022 Dec 4;93(2):437–52. doi: 10.1111/bjep.12567 (PMC10952248; doi:10.1111/bjep.12567)
Supplement: Supplementary file 1 — Appendix S1. [file BJEP-93-437-s001.docx]

**Teachers’ Motivational Prosody: A Pre-Registered Experimental Test of Children’s Reactions to Tone of Voice Used by Teachers**

**Supplemental Materials: Stimuli used and validation process**

**Stimuli.** Auditory samples were pre-recorded by teachers through a project-specific website audio recorder. Teachers were recruited on Prolific Academic and informed that they would be participating in a study that explores the role of teachers’ voice use in the classroom. Out of a larger set of voice recordings, seven speakers (3 male, age range 22 - 48 years (*M* = 37.6) all trained as teachers) who had pre-recorded a full set of 45 sentences in three different ways were selected to be played to children in this study. Teachers received written instructions before the recordings took place. These outlined under which circumstances teachers might use each voice. For neutral sounding stimuli they were told to use a “regular, what we call ‘centered neutral’, teaching voice. We think you will speak in your natural, optimum pitch and situations in which you might use it, may include making announcements, introductions, or demonstrations”. For autonomy-supportive recordings, “we are asking you to imagine that you are addressing a child in your class that is in need of praise (e.g. for good behaviour), encouragement (e.g., to try a new task), or support (e.g., after a disagreement with another child). We call the voice that you typically use to speak in such situations the ‘autonomy-supportive’ voice; it is a voice that you use when providing children with meaningful choices and that communicates no external pressure.” For stimuli to be read in a controlling sounding voice, “we are asking you to imagine that you are addressing a child in your class who needs to pay more attention or needs to be disciplined. They may have engaged in unacceptable behaviour or have heard multiple warnings. We call this voice the ‘controlling’ voice; it is a firm voice that communicates a sense of pressure (e.g., you demand that the child does something).” Thus, each teacher recorded three sets of 45 sentences identical in content speaking in a neutral, autonomy-supportive, or controlling sounding voice. For the current manipulation, we selected 11 semantically neutral sentences to be played to children (all content relevant to the school day, including “I’m waiting for people to quiet down”, “It is time to tidy up all of your belongings”, and “I need your attention”). Individual sentences were concatenated using praat (Boersma & Weenink, 2021) with a short gap in between sentences to create 80 second long sound clips. Tone of voice varied across conditions (see Rogerson & Dodd, 2005 for similar approach). Sentence content was repeated within a concatenated file, but not spoken by the same speaker. Materials are available upon request.

**Stimuli validation**. Stimuli used were acoustically analyzed using Praat (Boersma & Weenink, 2021). Descriptive results can be found in Table 1. In line with previous research (e.g., Weinstein et al., 2018), values suggest that pitch was higher for motivational as opposed to neutral prosody, with the highest pitched utterances observed when using an autonomy-supportive voice. Motivational prosody was also marked with slower speech rate compared to neutral. Controlling sounding voices were spoken slightly faster than autonomy-supportive sounding voices. Crucially, as a marker of voice quality, energy present in higher frequency bands is higher when looking at controlling sounding voices compared to autonomy-supportive and neutral voices. The latter acoustic marker has repeatedly been argued to lead to the perception of effortful, harsh sounding voices, critical indicators of pressuring, demanding sounding speech (Scherer, 1986; Weinstein et al., 2018).

To further validate materials, we conducted a perceptual rating study with 30 participants. Participants were between 18-24 years old (*M* = 20.60), and 20 of them were female. They were asked to listen to the concatenated files and rate the extent to which the voices in each clip sounded pressuring (1 = *not at all pressuring* to 7 = *extremely pressuring*) and the extent to which they sounded autonomy-supportive (1 = *does not support choice* to 7 = *extremely supportive of choice*). Results from paired samples *t*-tests showed that stimuli in the autonomy-supportive condition were rated as supporting a greater sense of choice (*M* = 4.07; *SD* = 1.72) than did the controlling condition (*M* = 1.72; *SD* = 1.26, *t*(29) = 7.04, *p* < .001, *d* = 1.83); the controlling condition was rated as more pressuring (*M* = 6.24; *SD* = 1.13) than the autonomy-supportive condition (*M* = 2.76; *SD* = 1.59; *t*(29) = -2.81, *p* < .001, *d* = 1.81). Neutral sounding stimuli were rated closer to the midpoints, sounding neither particularly pressuring (*M* = 3.55; *SD* = 1.79) nor supportive of choice (*M* = 2.90; *SD* = 1.48). This condition was lower on perceived choice than the autonomy-supportive condition, *t*(29) = 3.82, *p* < .001, *d* = 1.68, and lower on perceived pressure than the controlling condition, *SD* = 1.78, *t*(29) = 8.26, *p* < .001, *d* = 1.78. Taken together, both acoustic and perceptual validation analyses showed that teachers’ intention to convey a specific motivational voice pattern was successfully perceived by young adults.

**Table 1**

*Acoustic Indicators Within Each of the Three Conditions (left column)*

|  | Mean F0 (Hz) | Mean Intensity (dB) | Speech rate (words/sec) | Low frequency band (dB) | High frequency band (dB) |
| --- | --- | --- | --- | --- | --- |
| Autonomy-supportive | 220.4 | 75.6 | 3.7 | 43.9 | 24.9 |
| Control | 214.2 | 75.7 | 3.5 | 42.6 | 29.9 |
| Neutral | 174.8 | 76.0 | 3.9 | 44.1 | 25.3 |

*Note.* Acoustic measures were extracted from individual files that were included in the 80 second long concatenated files. We extracted measures for perceived pitch (fundamental frequency, F0), loudness (intensity), speech rate (number of words uttered per second) and - as indicators for perceived roughness of a voice - we calculated the relative amounts of energy for a low (0-1000Hz) and high (2000-5000Hz) frequency band range.

**Figures 1a-1c** show example waveforms for the same sentence spoken in different prosodies (Fig 1a. shows neutral; Fig 1b. shows autonomy-supportive; Fig 1c. shows controlling prosody). Under each waveform, superimposed on the spectrogram, pitch contours are plotted to show how pitch was modulated over time. The darker shades in the spectrogram indicate increased energy use of speakers.

Figure 1a: neutral condition example.
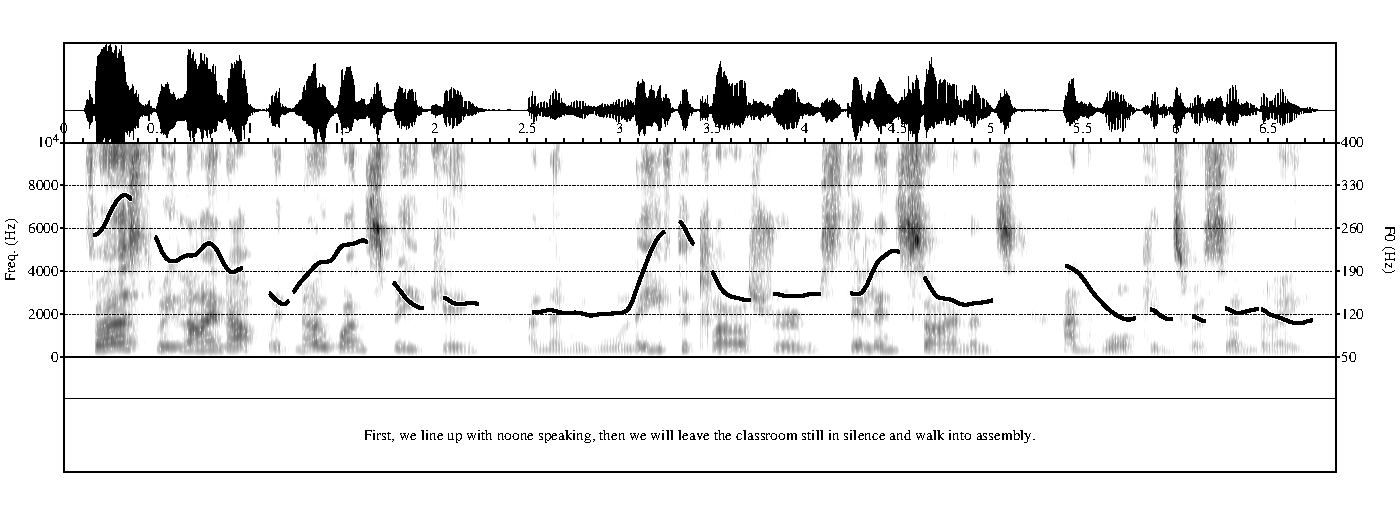


Figure 1b: autonomy support condition example.


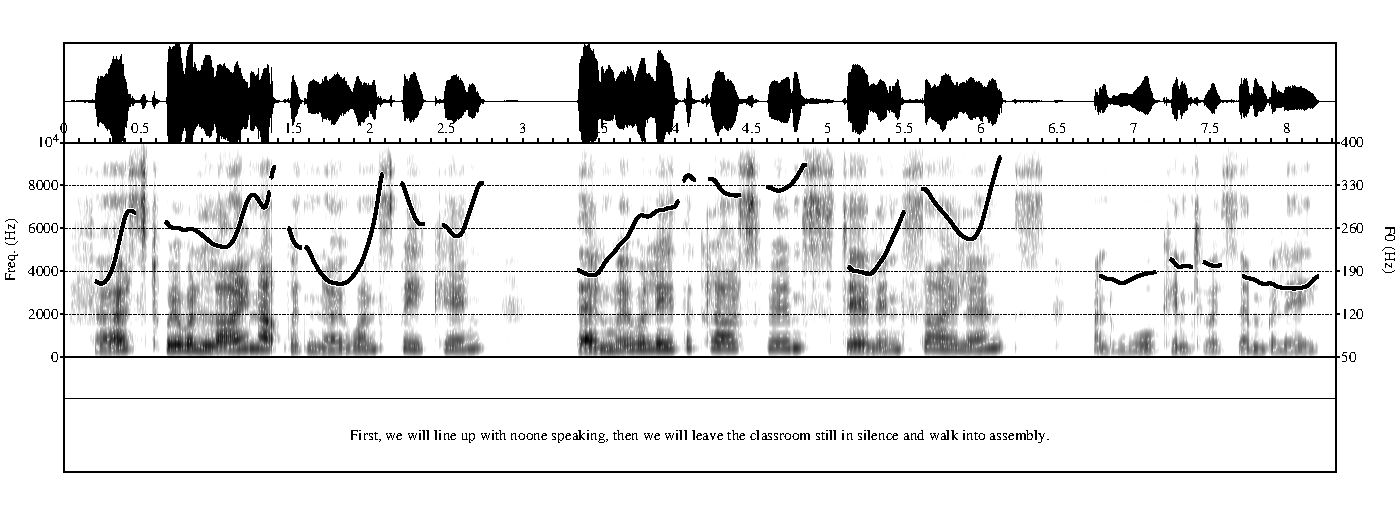


Figure 1c: control condition example.


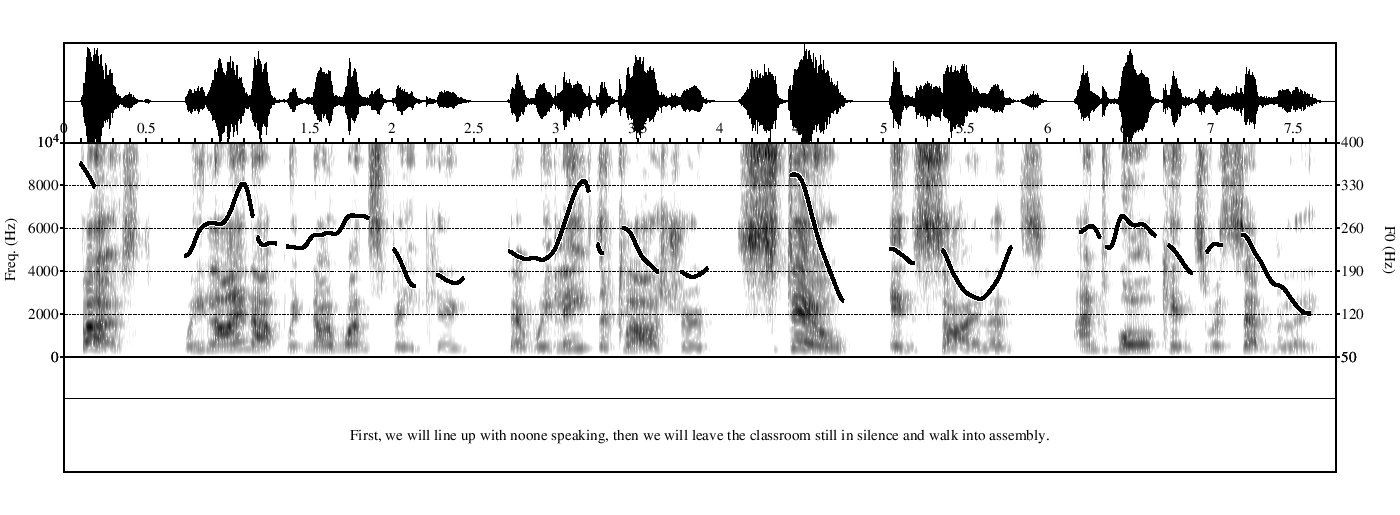


**Reference:**

Boersma, P., & Weenink, D. (2021). Praat: Doing phonetics by computer [Computer program]. Version 6.1.50. http://www.praat.org/
